# Hereditary Transthyretin Amyloidosis in Austria: Clinical, Genetic, and Demographic Insights from a Nationwide Cohort

**DOI:** 10.3390/jcm15051958

**Published:** 2026-03-04

**Authors:** Nikita Ermolaev, Wolfgang N. Löscher, Nicolas Verheyen, Gerhard Pölzl, Klemens Ablasser, Hermine Agis, Christina Binder, Diana Bonderman, Hakan Cetin, Franz Duca, Theresa Antonia Griedl, Sandra Hacker, Viktoria Höller, Andreas Kammerlander, Lukas Kellermair, Vera E. A. Kleinveld, Christina Kronberger, Deddo Mörtl, Michael Poledniczek, Christian Reiter, Rene Rettl, Lena Marie Schmid, Nora Schwegel, Elisabeth Schaumberger, Raute Sunder-Plassmann, Maria Ungericht, Reinhard Windhager, Fritz Zimprich, Roza Badr Eslam, Michaela Auer-Grumbach

**Affiliations:** 1 Department of Clinical Pharmacology, Medical University of Vienna, 1090 Vienna, Austria; nikita.ermolaev@meduniwien.ac.at; 2 Department of Neurology, Medical University of Innsbruck, 6020 Innsbruck, Austria; 3 Department of Cardiology, University Heart Center Graz, 8036 Graz, Austria; 4 Department of Cardiology, Medial University of Innsbruck, 6020 Innsbruck, Austria; 5 Department of Hematology & Hemostaseology, Medical University of Vienna, 1090 Vienna, Austria; 6 Department of Cardiology, Medical University of Vienna, 1090 Vienna, Austria; 7 Medical Department 5, Clinic Favoriten, Internal Medicine and Cardiology, 1090 Vienna, Austria; 8 Department of Neurology, Medical University of Vienna, 1090 Vienna, Austria; 9 Department of Neurology, Medical University of Graz, 8036 Graz, Austria; 10 Department of Orthopaedics and Trauma Surgery, Medical University of Vienna, 1090 Vienna, Austria; 11 Department of Neurology, Medical Faculty, Kepler University Hospital, 4020 Linz, Austria; 12 Department of Internal Medicine 3, University Hospital St. Pölten, 3100 St. Pölten, Austria; 13 Department of Cardiology, Medical Faculty, Kepler University Hospital, 4020 Linz, Austria; 14 Department of Neurology, Barmherzige Brueder Hospital, 4020 Linz, Austria; 15 Department of Laboratory Medicine, Medical University of Vienna, 1090 Vienna, Austria

**Keywords:** ATTR amyloidosis, *TTR* variants, Austria, genetic screening, regional distribution

## Abstract

**Background/Objectives**: Hereditary transthyretin amyloidosis (ATTRv) is a heterogeneous multisystem disease caused by pathogenic transthyretin gene (*TTR*) variants. Increased awareness and availability of disease-modifying therapies have resulted in increased diagnoses, even in previously nonendemic regions. The aim of this study was to update the nationwide Austrian ATTRv registry by characterizing the clinical, genetic, and regional distribution of *TTR* variants. **Methods**: This multicenter, observational analysis examined ATTRv cases diagnosed in Austria between 2014 and 2025. Individuals were included according to the presence of pathogenic or likely pathogenic variants or variants of uncertain significance (VUSs) in *TTR*. **Results**: In total, 100 individuals were identified, including symptomatic and asymptomatic carriers. Compared with our previously presented data, the number of genetically confirmed ATTRv cases has more than doubled. Twenty-three *TTR* variants were identified. The most frequent pathologic variants were p.His108Arg (26%), p.Ile127Phe (11%), and p.Thr69Ile (9%), while p.Val113Leu (9%) represented the most frequent VUS. Significant regional clustering of p.His108Arg was documented in Vienna and Lower Austria. Other findings included a rising number of p.Val142Ile carriers and phenotypically relevant VUSs in 20 patients. **Conclusions**: Our findings revealed an increasing detection rate of ATTRv in a nonendemic European region. These data underscore the importance of multidisciplinary evaluation, cascade testing, and long-term monitoring to improve early diagnosis and timely management in hereditary amyloidosis.

## 1. Introduction

Hereditary transthyretin amyloidosis (ATTRv) is a rare, progressive, multisystem disease of interdisciplinary interest caused by pathogenic transthyretin gene (*TTR*) variants [1]. It is a part of broader group of systemic amyloidoses, characterized by the extracellular deposition of misfolded amyloid fibrils in multiple tissues and organs. In ATTRv, these deposits most commonly affect the peripheral nervous system and heart; however, clinical involvement can extend to the autonomic nervous system, gastrointestinal tract, kidneys, and other organs [2,3]. The clinical presentation of ATTRv is heterogeneous and largely influenced by the underlying variant, with phenotypes ranging from predominantly neuropathic to cardiac or mixed forms [4].

Diagnosing systemic amyloidosis remains challenging because of its nonspecific but progressive symptoms and overlapping features with more common conditions such as chronic inflammatory demyelinating polyneuropathy and other forms of late-onset neuropathies, heart failure with preserved ejection fraction, hypertrophic cardiomyopathy, and hypertensive heart disease [5,6,7]. This diagnostic complexity is particularly evident in ATTRv, which is often misdiagnosed or only diagnosed at advanced stages. However, recent years have witnessed significant advances in the diagnostic landscape; specifically, noninvasive imaging (e.g., bone scintigraphy), biomarker-based approaches, and genetic testing have improved diagnostic accuracy and access [8].

In parallel, growing awareness of *TTR* amyloidosis, together with the implementation of disease-modifying therapies, including *TTR* stabilizers, RNA interference therapies, antisense oligonucleotides, and investigational therapies such as CRISPR-Cas9 technology and monoclonal antibodies, has transformed ATTRv from a previously palliative disease to a treatable condition [9,10]. These developments have contributed to substantial rise in reported *TTR* diagnoses over the last decade, transforming *TTR* amyloidosis from rarely recognized condition into increasingly identified and treatable disease [11,12].

Because of its autosomal dominant inheritance, ATTRv often affects multiple members within a family, and it might be under-recognized in asymptomatic relatives. In response to these patterns, several centers have developed nationwide diagnostic strategies and cascade screening programs to facilitate early detection and improve clinical outcomes [13,14,15,16].

In Austria, we previously reported the nationwide prevalence of ATTRv and the distribution of *TTR* variants [17]. Subsequently, diagnostic capabilities and interdisciplinary cooperation have continued to evolve, and the number of diagnosed individuals has substantially increased.

In the present study, we present a comprehensive 5-year update of the incidence of ATTRv in Austria, aiming to describe the clinical and genetic spectrum, explore the regional distribution of variants, and highlight the developing diagnostic landscape to support continuous improvements in awareness and personalized management of ATTRv.

## 2. Materials and Methods

### 2.1. Setting and Study Design

In this multicenter, observational cohort study, the dataset was compiled through the collaboration of multiple clinical centers located in different Austrian federal provinces, all of which participate in the diagnostic evaluation and long-term management of patients with suspected or confirmed ATTRv. The present study was framed as a clinical registry (EC#1079/2023 and #1738/2012), and the study protocol complied with the Declaration of Helsinki. All patients provided written informed consent prior to inclusion in this study, and patients underwent prospective follow-up.

### 2.2. Study Population

The present analysis was based on an expanded Austrian ATTRv registry, which was established in 2014. The registry was updated between January 2020 and July 2025 through continued collaboration with regional neurology and cardiology centers. During this period, we systematically included newly identified individuals with confirmed *TTR* variants.

The current analysis included patients and asymptomatic patients, all of whom were confirmed to carry a *TTR* variant, as determined via accredited molecular genetic testing. Patients with pathogenic and likely pathogenic variants as well as those carrying variants of uncertain clinical significance (VUSs) were eligible for inclusion. In addition, patients with ATTRv who died between January 2020 and July 2025 were included.

Patients were primarily referred for genetic testing if they presented with either hypertrophic cardiac phenotype of unclear etiology or progressive peripheral neuropathy. Moreover, in selected families, at-risk family members were genetically tested after providing informed consent. All probands were subjected to multidisciplinary evaluation at specialized clinics. The final cohort comprised all genetically confirmed *TTR* variants carriers recorded in the Austrian ATTRv registry between 2014 and July 2025, integrating cases from the original registry period (with the exception of those who had died before 2020) as well as additional individuals identified during the phase between January 2020 and July 2025.

### 2.3. Diagnostic Work-Up

Diagnostic evaluation for ATTRv was conducted through a multidisciplinary approach and focused on confirming both organ involvement and genetic etiology. Two main clinical pathways were followed according to the predominant presentation: suspected cardiac amyloidosis and suspected amyloid-mediated neuropathy.

#### 2.3.1. Cardiac Amyloidosis Evaluation

In patients with unexplained left ventricular hypertrophy or red-flag findings (e.g., low voltage on ECG, discordant voltage-to-mass ratio, bilateral carpal tunnel syndrome [CTS]), cardiac amyloidosis was assessed using a structured diagnostic algorithm, as previously suggested by the ESC Working Group on Myocardial and Pericardial Diseases [18]. The work-up included echocardiography, cardiac magnetic resonance imaging when indicated, and bone scintigraphy with [^99m^Tc]-labeled tracers to detect *TTR*-related amyloid deposition. Light chain amyloidosis was excluded through serum and urine immunofixation and free light chain analysis. A diagnosis of transthyretin cardiac amyloidosis was established in the presence of grade 2 or 3 cardiac uptake on bone scintigraphy in the absence of monoclonal gammopathy or through bioptic confirmation in certain cases.

#### 2.3.2. Amyloid-Mediated Neuropathy Evaluation

All patients and at-risk family members were evaluated neurologically using standard procedures. Red flags included progressive length-dependent axonal neuropathy and/or autonomic dysfunction, bilateral CTS, gastrointestinal dysmotility, unexplained weight loss, and family history of neuropathy or amyloidosis. Nerve conduction velocity studies followed standard techniques for stimulation and recording. Laboratory tests were performed to exclude common causes of polyneuropathy (PNP; e.g., diabetes, renal failure, alcohol, B12 deficiency, and elevated free light chain levels, among others).

#### 2.3.3. Genetic Testing and Classification of TTR Variants

Genetic testing and counseling were performed following established protocols [19]. However, notably, the allocation of testing was not fully standardized as patients were recruited from various institutions across Austria and analyses were performed in different diagnostic laboratories. In most cases, genetic testing was conducted once at the time of initial evaluation. The ClinVar and Genome Aggregation Database databases were used to assess the impact of the detected variants.

### 2.4. Statistical Analysis

All statistical analyses were performed using SPSS version 29.0 (IBM, Armonk, NY, USA). Categorical variables are presented as counts and percentages, whereas continuous variables are presented as mean ± standard deviation or median with interquartile range depending on the data distribution. The study was designed as a descriptive registry update and was not powered or structured to perform formal comparative, prognostic, or survival analysis.

## 3. Results

### 3.1. Overview of the Study Cohort

**Figure 1:**
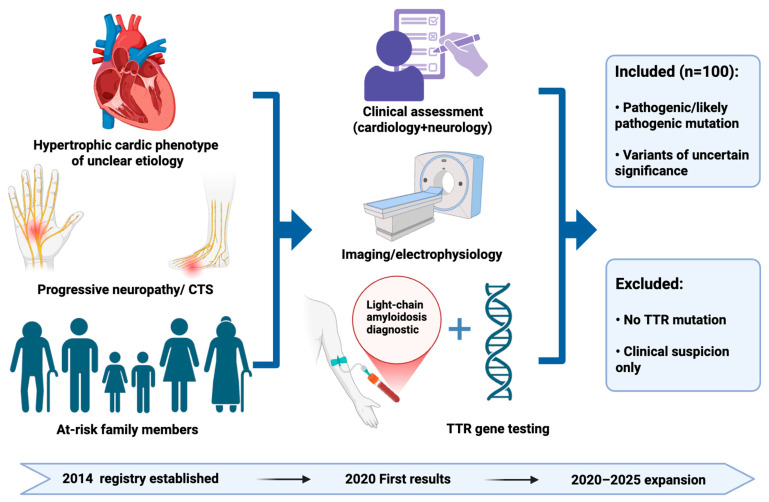
Diagnostic flow and inclusion pathway of the Austrian ATTRv cohort. Individuals presenting with a hypertrophic cardiac phenotype, progressive neuropathy, or family history underwent structured multidisciplinary evaluation, including imaging and/or biopsy, electrophysiology, and laboratory and genetic testing. Only individuals with confirmed *TTR* variants (pathogenic, likely pathogenic, or variants of uncertain significance) were included in the final cohort. The registry was initiated in 2014 and expanded between January 2020 and July 2025. Abbreviations: CTS, carpal tunnel syndrome; *TTR*, transthyretin. This figure was created using BioRender.com.

**Figure A1:**
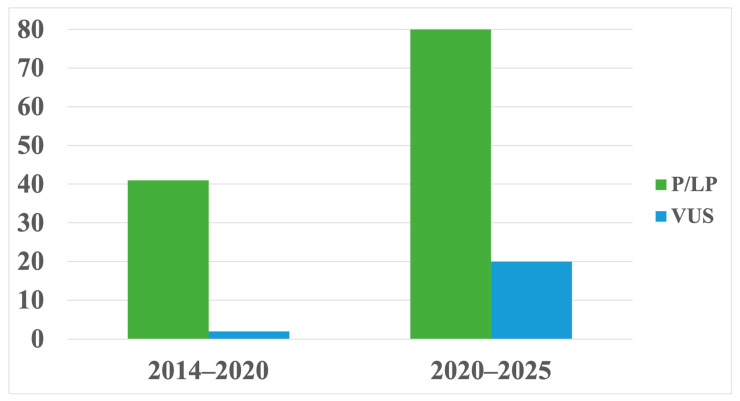
Descriptive overview of newly diagnosed ATTRv individuals across registry periods (2014–2020 vs. 2020–2025). Bars indicate the number of individuals carrying pathogenic/likely pathogenic (P/LP) variants and variants of uncertain significance (VUSs).

During the study period, 100 individuals (41 women and 59 men; mean age at the time of diagnosis: 58.2 ± 15.7 years) from 55 families were included as carriers of pathogenic/likely pathogenic variants or VUSs in *TTR*. A descriptive overview of ATTRv diagnoses across is shown in Figure A1. Figure 1 presents a schematic overview of the study cohort inclusion process.

**Figure 2:**
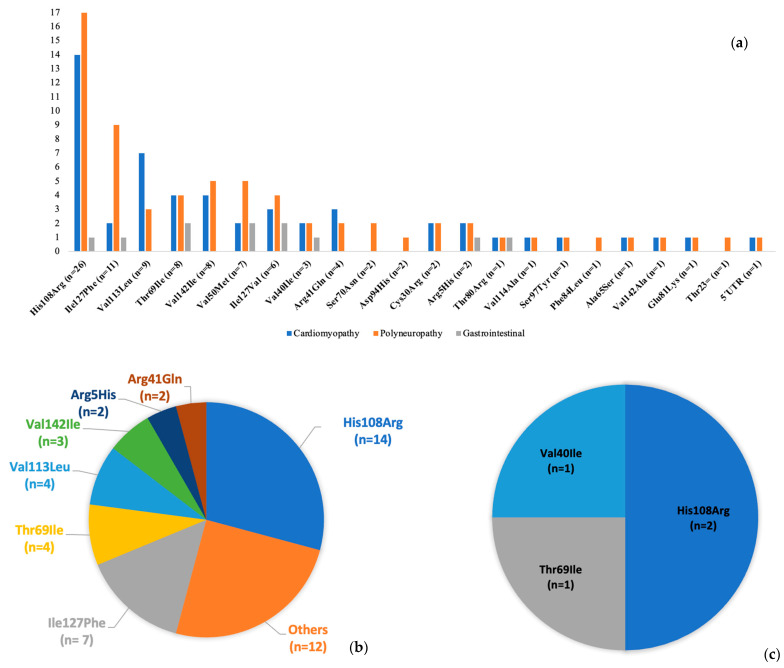
(**a**) Phenotypic distribution across *TTR* variants. Each variant is annotated with the total number of individuals on the X-axis, and the Y-axis presents the number of patients who presented with each phenotype. Importantly, multiple phenotypes can co-occur within a single patient. p.Glu109Gln is not depicted in the chart, as this individual was asymptomatic. (**b**) This figure illustrates the distribution of carpal tunnel syndrome among *TTR* variants. All variants that were observed in one individual each were grouped under “Others,” and included p.Val50Met, p.Thr80Arg, p.Val114Ala, p.Val40Ile, p.Ser70Asn, p.Asp94His, p.Ile127Val, p.Phe84Leu, p.Glu81Lys, p.Cys30Arg, p.Thr23= and a 5′UTR variant. (**c**) This figure presents the number of patients who underwent heart transplantation stratified by the underlying *TTR* variant. Abbreviation: *TTR*, transthyretin.

Among all individuals included, 72 were symptomatic at the time of testing, whereas the remaining 28 were asymptomatic carriers. Clinical manifestations varied across the cohort. In total, 52 individuals presented with cardiomyopathy, which is typically characterized by concentric hypertrophy, diastolic dysfunction, and conduction abnormalities. The most common neurological manifestation, being observed in 67 individuals, was length-dependent axonal sensorimotor peripheral neuropathy, often accompanied by autonomic features. Gastrointestinal symptoms, such as unexplained weight loss, diarrhea, and gastrointestinal dysmotility, were not systematically assessed, but they were reported by approximately 11% of the patients in the cohort. The distribution of clinical phenotypes among symptomatic individuals is summarized in Figure 2a, which illustrates the relative frequency of cardiac, neurologic, and gastrointestinal involvement within the cohort. Forty-eight probands had a history of CTS, which predated the diagnosis of ATTRv by several years in most cases. The distribution of CTS among *TTR* variants is illustrated in Figure 2b.

In a subset of patients, the disease progressed to advanced heart failure symptoms requiring heart transplantation. Four patients underwent heart transplantation during the observation period. The distribution of heart transplantation cases by variant type is presented in Figure 2c. Twelve patients carrying the *TTR* variants p.His108Arg (n = 6), p.Arg41Gln (n = 2), p.Val114Ala (n = 1), p.Ile127Phe (n = 1), p.Val113Leu (n = 1) and p.Ile127Val (n = 1) died during the observational period, either due to cardiac or gastrointestinal complications.

### 3.2. Genetic Spectrum of TTR Variants

**Figure 3:**
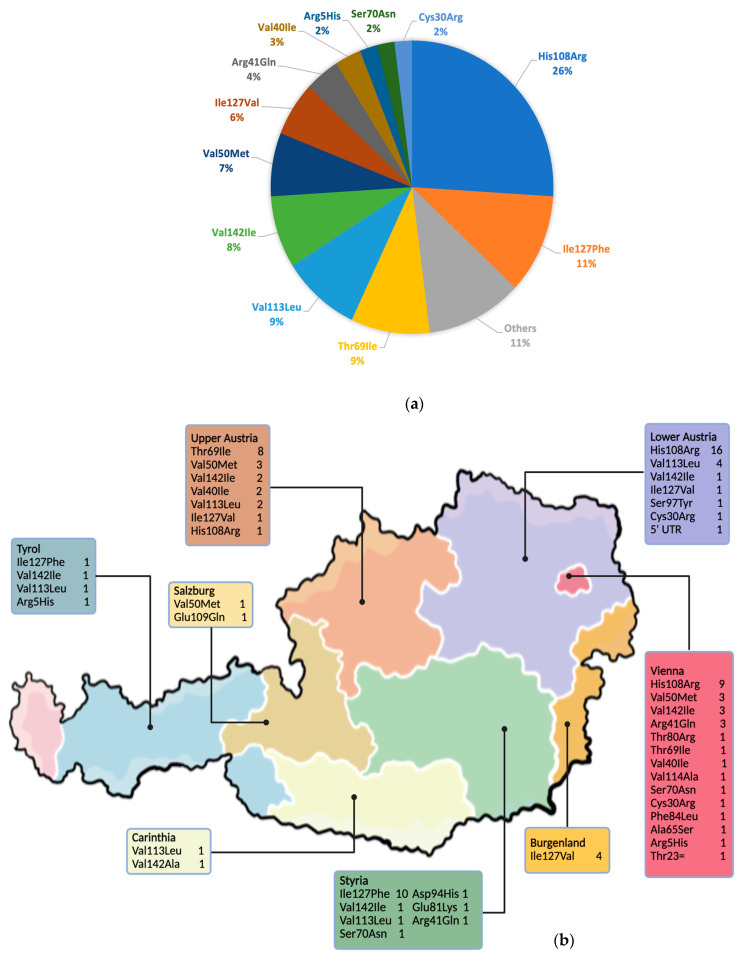
(**a**) Distribution of *TTR* variants in the Austrian ATTRv cohort. The most frequent variant was p.His108Arg, accounting for 26% of all cases, followed by p.Ile127Phe (11%), p.Thr69Ile (9%), p.Val113Leu (9%) and p.Val142Ile (8%). Less common variants included p.Val50Met (7%) and p.Ile127Val (6%). Rare variants, including p.Ala65Ser, p.Thr80Arg, p.Glu81Lys, p.Phe84Leu, p.Asp94His, p.Ser97Tyr, p.Glu109Gln, p.Val114Ala, p.Val142Ala, p.Thr23= and a 5′UTR variant, were grouped under “Others” (11%). (**b**) The map illustrates the regional spread of ATTRv cases by federal state. Each box lists the *TTR* variants detected in that region along with their respective case counts. This figure was created using freepik.com. Abbreviations: *TTR*, transthyretin; ATTRv, hereditary transthyretin amyloidosis.

In total, 23 distinct *TTR* variants were identified across the study cohort. The most frequently observed variant was p.His108Arg, which was detected in 26% of the patients, followed by p.Ile127Phe (11%), p.Thr69Ile (9%), p.Val113Leu (9%), p.Val142Ile (8%) and p.Val50Met (7%). Rare variants, each observed in a single individual, were collectively observed in 11% of the patients in the cohort. The relative distribution of all *TTR* variants is presented in Figure 3a.

The distribution of all *TTR* variants across the nine federal states of Austria is presented in Figure 3b. The largest proportion of variant carriers was identified in Vienna, followed by Lower Austria and Upper Austria, where several regional clustering patterns were observed. p.His108Arg, the most common variant, was predominantly detected in patients from Vienna and Lower Austria, consistent with the founder effect confirmed by genealogical and genetic linkage studies [20]. The p.Thr69Ile and p.Val113Leu variants were most frequently observed in Upper Austria, whereas rarer variants were scattered across other regions, including Tyrol, Styria, and Carinthia.

**Figure A2:**
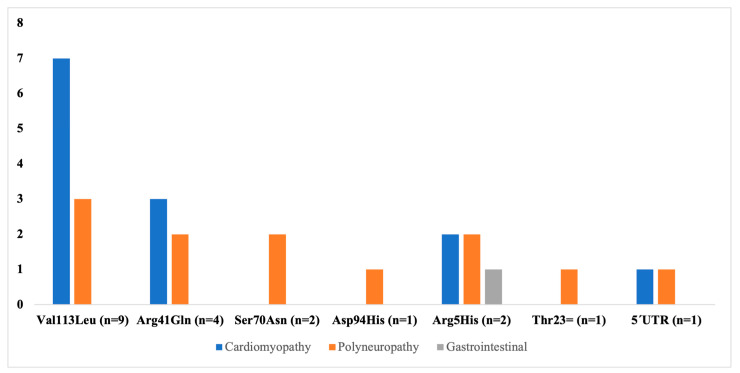
Phenotypes of patients with variants of uncertain significance.

In addition to clearly pathogenic and likely pathogenic variants, 20 individuals in the cohort carried VUSs based on available entries in ClinVar. These variants included p.Arg41Gln (n = 4, exon 2, c.122G>A), p.Arg5His (n = 2, exon 1, c.14G>A), p.Ser70Asn (n = 2, exon 3, c.209G>A), p.Asp94His (n = 1, exon 3, c.280>C), p.Val113Leu (n = 9, exon 4, c.337G>C), the synonymous variant p.Thr23= (n = 1, exon 1, c.69G>C) and one 5′UTR variant (c.61G>A). Most of these individuals presented with clinically relevant symptoms. Cardiomyopathy, PNP, and gastrointestinal symptoms were observed in 13, 12, and 2 patients, respectively. All individuals carrying VUSs were phenotypically characterized based on non-invasive imaging modalities and clinical investigations. Histopathological confirmation was not routinely performed in this cohort. Detailed information about the phenotypes of patients with VUSs is presented in Figure A2. Notably, p.Cys30Arg was listed as “pathogenic/VUS” in databases, and both p.Glu81Lys and p.Phe84Leu were classified as “pathogenic/likely pathogenic,” reflecting an intermediate level of evidence.

### 3.3. Regional and Ancestral Origin of Patients

In addition to the geographic distribution within Austria, patients differed in their ancestry, reflecting the country’s demographic diversity. Most individuals were of Austrian origin, with several individuals having ancestry from other countries, including Tunisia, Senegal, Nigeria, Syria, Iraq, Egypt, Macedonia, Italy, Israel, Hungary, Slovakia, Germany, and Pakistan. For instance, p.Val142Ile was observed in patients with Austrian, North African, and West African ancestry; p.Thr80Arg was detected in a patient of Iraqi descent; and p.Val50Met was found in individuals of Austrian, Pakistani, and Slovakian descent.

Although the most prevalent variants such as p.His108Arg, p.Thr69Ile and p.Ile127Phe were predominantly detected in Austrian individuals, several rarer variants appeared to be ethnically or geographically linked, suggesting either migration-associated introduction or under-recognized regional founder variants. The full list of variants with their classification is provided in Table 1.

## 4. Discussion

Through this nationwide cohort-based study, we present a comprehensive update on the phenotypic, genetic, and demographic characteristics of ATTRv in Austria. During the initial registry period from 2014 to 2020, 43 genetically confirmed ATTRv cases were identified and reported previously. In this subsequent update period from January 2020 to July 2025, an additional 57 individuals were diagnosed. Based on the current number of cases (n = 100) and the Austrian population of approximately 9.1 million, the estimated prevalence of ATTRv in Austria is approximately 1.1 per 100,000 inhabitants, representing a 2-fold increase compared with our 2020 estimate of approximately 1 per 200,000 inhabitants [17]. However, ATTRv remains a rare disease, and its prevalence varies widely across Europe.

In addition, recent national registries of other countries documented an increasing prevalence, particularly in previously nonendemic countries such as Italy. For example, a multicenter Italian survey reported 373 active ATTRv cases as of March 2024, corresponding to a prevalence of approximately 6.3 per million, representing a 50% increase compared with earlier estimates [21,22]. Similar trends have been observed in other non-endemic European populations, including the United Kingdom [23]. Together, these European data indicate that ATTRv in nonendemic regions may have been substantially under-recognized in the past, but the implementation of diagnostic algorithms has significantly reshaped the epidemiological landscape. Conversely, historically endemic regions such as Portugal or Sweden continue to display higher prevalence rates, predominantly driven by the p.Val50Met variant (historically termed p.Val30Met) [24,25,26]. Interestingly, we identified four new carriers of the p.Val50Met variant, but this variant remains rare in Austria, being present in only 7% of our cohort. Notably, the Austrian p.Val50Met carriers in our cohort had diverse ancestral backgrounds, including individuals with Pakistani and Slovakian descent, suggesting migration-associated introduction rather than a local founder effect.

The most frequent variant in our population was p.His108Arg, which primarily clusters in Vienna and Lower Austria, consistent with a known regional founder effect [20]. Interestingly, other recurrent variants such as p.Thr69Ile, p.Val113Leu and p.Ile127Phe exhibited geographic clustering, suggesting potential under-recognized founder effects and having direct implications for targeted screening strategies. Compared with previous data, the diversity of variants has substantially expanded, with 23 different variants (16 pathogenic/likely pathogenic variants and 7 VUSs) being identified, including several rare and ethnically linked variants. The increasing number of individuals carrying p.Val142Ile is of particular interest. While only one patient carrying p.Val142Ile was identified up to 2020, this number increased to eight in this analysis. Importantly, four of these individuals (50%) were of Austrian origin, suggesting either a broader geographic spread of this variant or an under-recognized presence in the local population. p.Val142Ile is a well-known variant that is predominantly associated with transthyretin cardiac amyloidosis, especially in individuals of African ancestry, as it has been found in up to 3.5% of the African population [27]. However, recent data suggest that this variant also occurs in non-African individuals with variable penetrance and phenotypes, as highlighted by da Costa et al., who documented p.Val142Ile in Portuguese patients, emphasizing its relevance beyond traditionally recognized populations [28]. In addition, de Frutos et al. described the significant prevalence of this variant in the Spanish population [29]. In our Austrian cohort, phenotypic variability was evident among p.Val142Ile carriers. Four individuals presented with both cardiomyopathy and PNP, only one individual displayed isolated PNP, and three individuals remained asymptomatic at the time of data collection. These findings underscore the need to consider p.Val142Ile-associated disease beyond traditionally recognized high-risk population, particularly in cardiology settings.

The impact of VUSs, which were classified according to the information provided by the ClinVar database, remains a critical yet challenging aspect of ATTRv diagnostics. It must be clearly stated here that pathogenicity for each VUS will only be confirmed by detection of amyloid deposit in tissues. Although VUSs do not meet formal criteria for pathogenicity, they often appear in patients with compatible clinical phenotypes, positive family histories, or suggestive regional clustering. In our cohort, 20 individuals carried VUSs, all of whom were referred to genetic testing based on clinical relevance and multidisciplinary interpretation. The frequent presence of cardiomyopathy and/or PNP underscores the need to monitor such cases systematically. Accurate documentation and publication of such cases are essential to support future reclassification efforts, international database comparison, and segregation analysis, particularly in rare diseases in which functional data may be lacking. As genotype–phenotype correlations become more refined and larger datasets are aggregated, current VUSs could eventually become actionable variants. Finally, the repeated occurrence of VUSs, as observed for p.Val113Leu, underscores their potential clinical relevance and highlights the importance of systematic documentation and longitudinal follow-up, which may contribute to future reclassification when combined with phenotypic consistency and population-based evidence. Overall, our findings reflect the transition of ATTRv from a just rarely recognized hereditary PNP to an increasingly diagnosed systemic disorder in Europe.

## 5. Limitations

This study had some limitations. Despite progress in diagnostics, the true prevalence of ATTRv in Austria is likely underestimated. Because of incomplete penetrance and variable onset, many carriers possibly remain undiagnosed, particularly older individuals or those presenting with isolated cardiac symptoms or CTS, in whom ATTRv can mimic more common conditions. Cascade testing has facilitated earlier detection in affected families; however, systematic family screening was implemented in only few of our ATTRv families. In addition, genetic testing was not fully standardized across all individuals as they were recruited from different regions and genetical analyses were performed in multiple accredited diagnostic laboratories. With the increasing application of genetic sequencing in neurology and cardiology workflows, incidental detection of *TTR* variants is expected to increase, bringing new challenges, primarily in the management of asymptomatic individuals and those carrying VUSs.

## 6. Conclusions

In summary, this nationwide update of our ATTRv registry revealed a significant increase in diagnosed *TTR* variant carriers and highlighted the importance of national registries, long-term clinical follow-up, and genotype–phenotype correlation studies to inform early intervention strategies, assess penetrance, and better estimate the true epidemiological footprint of ATTRv in Central Europe.

## Figures and Tables

**Table 1:** Characteristics of *TTR* variants detected in the study cohort.

Exon	Consequence	c.DNA	SNP	ClinVar ID	Allele Frequency *	ClinVar	Nationality by Descendant
1	Arg5His	c.14G>A	rs138657343	181697	0.0001406	VUS	Austria
1	Ala65Ser	c.193G>T	rs121918078	850453	n/a	Pathogenic	Egypt
1	Thr23=	c.69G>C	n/a	640710	n/a	VUS	Austria
2	Cys30Arg	c.88T>C	rs121918083	13444	0.000001239	Pathogenic/VUS	Austria
2	Val40Ile	c.118G>A	rs121918093	13455	0.000001239	Pathogenic	Syria, Austria
2	Arg41Gln	c.122G>A	rs879254269	246463	0.000006815	VUS	Austria
2	Val50Met	c.148G>A	rs28933979	13417	0.00005700	Pathogenic	Austria, Pakistan, Slovakia
3	Thr69Ile	c.206C>T	rs1555631387	1454801	n/a	Pathogenic	Austria
3	Ser70Asn	c.209G>A	rs121918080	808383	0.00001921	VUS	Austria
3	Thr80Arg	c.238A>G	rs121918070	13421	n/a	Pathogenic	Iraq
3	Glu81Lys	c.241G>A	rs121918086	13447	6.195 × 10^−7^	Pathogenic/likely pathogenic	Austria
3	Phe84Leu	c.252T>G	rs2073510805	845368	6.195 × 10^−7^	Pathogenic/likely pathogenic	Italy
3	Asp94His	c.280G>C	rs730881164	181695	0.00005886	VUS	Austria
3	Ser97Tyr	c.290C>A	rs121918071	13422	0.000001859	Pathogenic	Israel
3	His108Arg	c.323A>G	rs2073511411	853918	n/a	Pathogenic	Austria, Hungary
3	Glu109Gln	c.325G>C	rs121918082	13442	n/a	Pathogenic	Macedonia
4	Val113Leu	c.337G>C	n/a	n/a	6.196 × 10^−7^	VUS	Austria
4	Val114Ala	c.341T>C	n/a	n/a	n/a	Pathogenic	Austria
4	Ile127Val	c.379A>G	rs121918089	13450	n/a	Pathogenic	Austria, Germany
4	Ile127Phe	c.379A>T	n/a	n/a	n/a	Pathogenic	Austria
4	Val142Ile	c.424G>A	rs76992529	13426	0.0008878	Pathogenic	Tunisia, Senegal, Austria, Nigeria
4	Val142Ala	c.425T>C	rs2144414426	1333466	n/a	Pathogenic	California
	5′UTR	c.61G>A	rs770403822	326544	n/a	VUS	Austria

Summary of all identified *TTR* variants, including their exon location, nucleotide changes, reference SNP ID, ClinVar classification, and geographic descent of carriers. Abbreviations: n/a, no data; VUS, variant of uncertain significance. * Frequency of *TTR* variants in the Genome Aggregation Database. Abbreviation: *TTR*, transthyretin.

## Data Availability

Data will be shared upon reasonable request to the corresponding author.

## Abbreviations

The following abbreviations are used in this manuscript:

ATTRv	Hereditary transthyretin amyloidosis
CTS	Carpal tunnel syndrome
PNP	Polyneuropathy
*TTR*	Transthyretin
VUS	Variant of uncertain significance
